# Cesarean section rates in the COVID-19 era: False alarms and the safety of the mother and child

**DOI:** 10.18332/ejm/134998

**Published:** 2021-05-20

**Authors:** Wissam Arab, David Atallah

**Affiliations:** 1 Department of Obstetrics and Gynecology, Hôtel-Dieu de France University Hospital, Saint-Joseph University, Beirut, Lebanon

**Keywords:** COVID-19, cesarean, preterm, vaginal delivery

**Dear Editor**,

The COVID-19 pandemic is ongoingly affecting numerous pregnant patients around the world. Meanwhile, nationwide lockdowns have drastically limited counseling and follow-up visits of women during pregnancy. Throughout these difficulties, delivering via the cesarean route is becoming common, both in the infected and non-infected pregnant population. Looking into pregnant women with COVID-19, around 64% delivered via a cesarean route^1^, with this rate reaching 93% in China^2^. Due to the relative state of immunosuppression in pregnancy, women might be at an increased risk of severe illness compared to non-pregnant women, after adjusting for age and other confounding factors^3^. Death has occurred in around 0.1% of the cases, with ICU admissions reaching 3%^4^. In China, the rate of severe disease was 8%^2^.

Knowing that the majority of COVID-19 infections in pregnancy occur in the third trimester^2,5^, with 15% requiring hospital admission^4^, the increased cesarean rate could be partially justified. However, many patients with non-severe disease are being delivered using the C-section route; experts consider that early delivery, even in non-severe cases, as beneficial for the subsequent treatment and outcome of COVID-19^16^. This has led to an increase in the rate of preterm deliveries (21%–31%), of which only few are related to spontaneous preterm labor and preterm premature rupture of membranes, while the rest are thought to be iatrogenic^5^. Preterm and term C-sections are performed due to concerns that excessive ventilation and stress during labor might aggravate the respiratory and pro-inflammatory status accompanying COVID-19^7^. Evidence also showed that maternal oxygenation can be quickly restored by delivery^6^. In parallel, new papers reported placental infection during the COVID-19, leading to placental vascular disease, preeclampsia-like syndrome, fetal growth restriction and higher risks of perinatal death^8^. Fearing these eventual adverse events could add to the increased rate of iatrogenic preterm deliveries using a C-section route. Also, some laboring women are undergoing C-sections due to prophylactic antiplatelets use against COVID-related thromboembolisms, precluding the administration of epidural anaesthesia; not forgetting the fear of vertical transmission in utero, which, even if yet unproven, is usually thought by obstetricians. This risk has been estimated to be around 5% and peaks at term^1,5^.

In regard to the pregnant population in general, fears of contracting COVID-19 at healthcare centers as well as difficulties in transportation during lockdown periods has contributed to a reluctance in timely referrals to emergency care units. These facts have led to a delay in the management of obstetrical complications and therefore to an increase in stillbirths and preterm deliveries, with a higher risk of C-section in this context^9^. On the other hand, some obstetricians are reluctant when it comes to exposing themselves, trainees and midwives to pregnant patients whose PCR status is unknown, with a tendency towards pursuing C-sections for laboring women who would normally have better chances for delivering vaginally; obstetricians lowered the C-section threshold during COVID-19 pandemic, with the aim of reducing inpatient maternal stays, cross-infection and the use of protective equipment^6^. Not forgetting, some pregnant women, worried about their partners not making it to the birth unit due to lockdowns and the unpredictability of labor, are choosing elective C-section for delivery.

We support the alarm raised in a previous study^10^, and challenge obstetric-led units in their tendency towards C-section. Favoring vaginal birth in infected women is paramount, as it decreases the risk of clinical deterioration, COVID-related thromboembolisms and neonatal morbidity related to iatrogenic preterm deliveries.
